# Supplementary material for: Computational Modeling of Drug Response Identifies Mutant-Specific Constraints for Dosing panRAF and MEK Inhibitors in Melanoma
Source: Cancers (Basel). 2024 Aug 22;16(16):2914. doi: 10.3390/cancers16162914 (PMC11353013; doi:10.3390/cancers16162914)
Supplement: Supplementary file 1 [file cancers-16-02914-s001.zip › cancers-3068963-supplementary.pdf]

Supplemental Materials

Table S1. Study Design for xenograft experiment.

Group	*No./ Sex*	*Treatment*	*Dose level (mg/kg)[a]*	*No./Sex*	*Route*	*Days of Dosing*	*Dose Conc. (mg/mL)[a]*	*Dose Volume (mL/kg)*
1	*10/F*	*Vehicles*	*0 (Vehicles)*	*10/F*	*PO*	*21*	*0*	*5, 5*
2	*10/F*	*Cobimetinib*	*5*	*10/F*	*PO*	*21*	*1.1*	*5*
3	*10/F*	*Belvarafenib*	*15*	*10/F*	*PO*	*21*	*3.3*	*5*
4	*10/F*	*Belvarafenib*	*30*	*10/F*	*PO*	*21*	*6.6*	*5*
5	*10/F*	*Belvarafenib + Cobimetinib*	*15 + 5*	*10/F*	*PO*	*21*	*3.3, 1.1*	*5, 5*
6	*10/F*	*Belvarafenib + Cobimetinib*	*30 + 5*	*10/F*	*PO*	*21*	*6.6, 1.1*	*5, 5*

Conc. = concentration; PO = orally; QD = once daily.

Note: Vehicle controls were 5% dimethyl sulfide/5% Cremophor EL (100 µ) + 0.5% (w/v) methylcellulose; 0.2% Tween 80™ (100 µL). Dose levels and concentrations are expressed as free-base equivalents and were dosed once daily (QD) for 21 days.

(a)

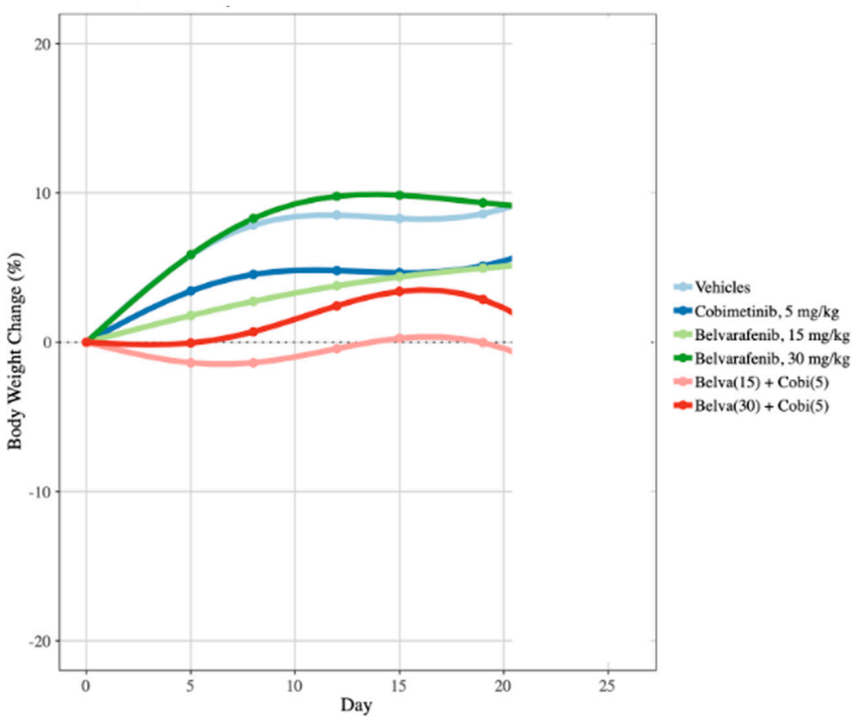

(b)

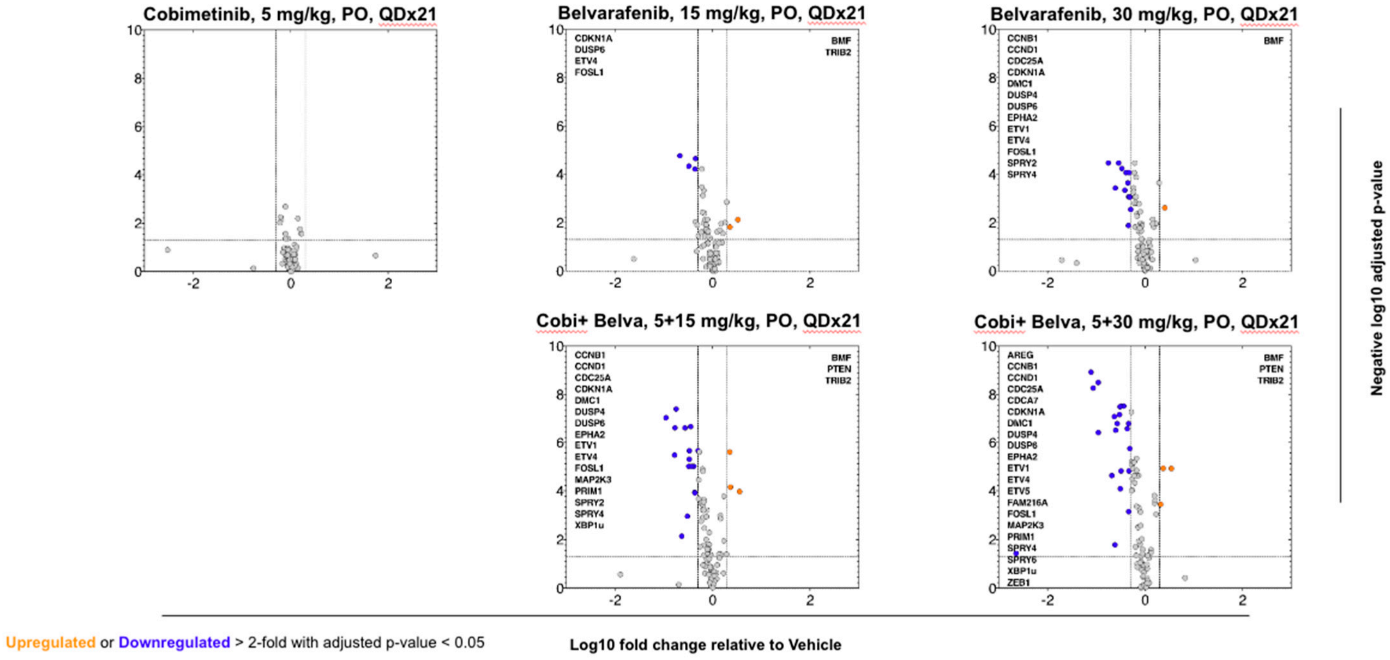

Figure S1. a) Body weight of mice in xenograft experiment. b) Gene expression response in IPC-298 xenografts at indicated times and doses of Belvarafenib and Cobimetinib. Upregulated or downregulated genes are marked in color and named on the sides. Three out of five conditions re-analyzed here were previously published in [25].

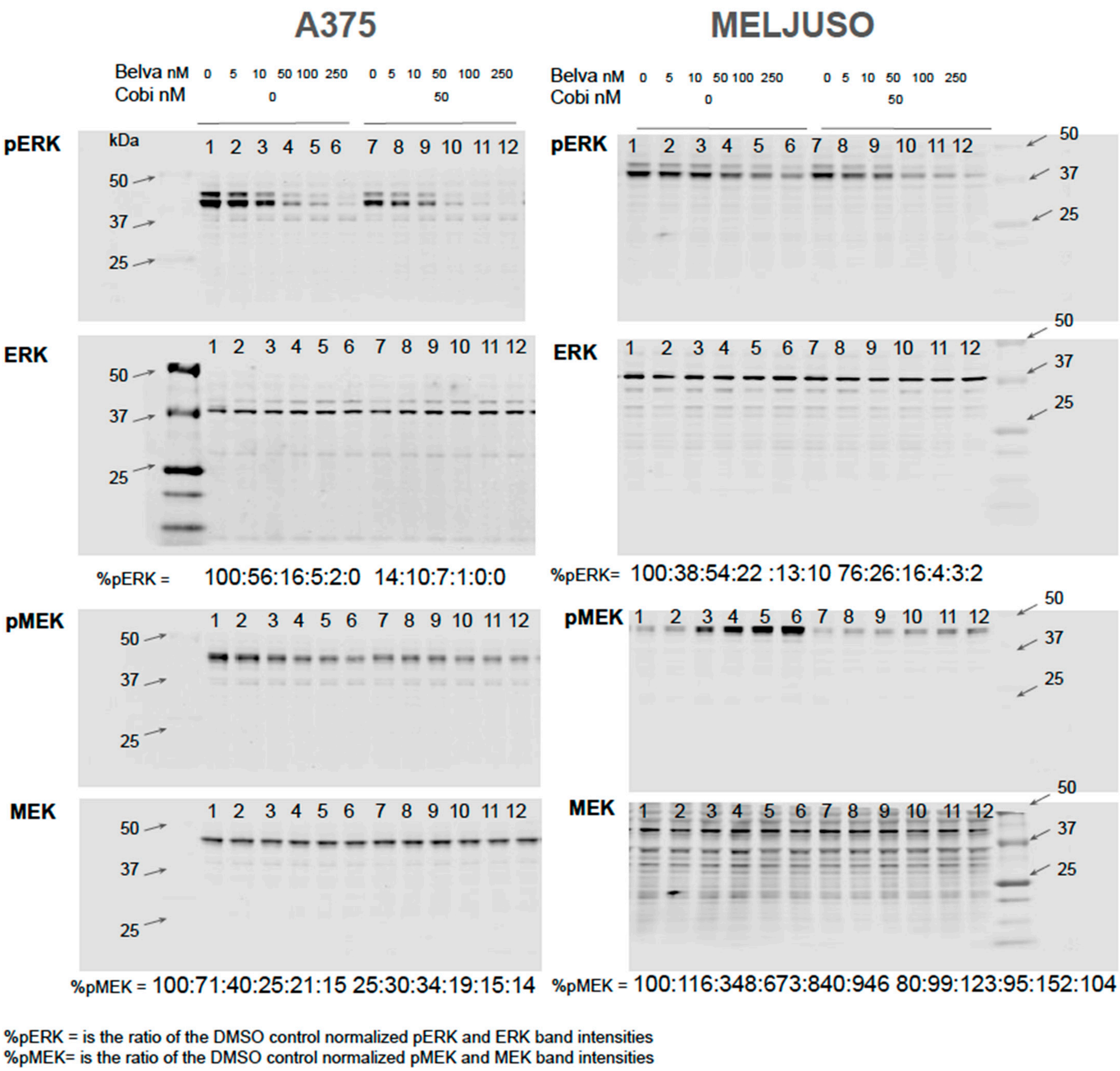

Figure S2. Uncropped Western Blots with quantifications related to Figure 2 (c-d). .

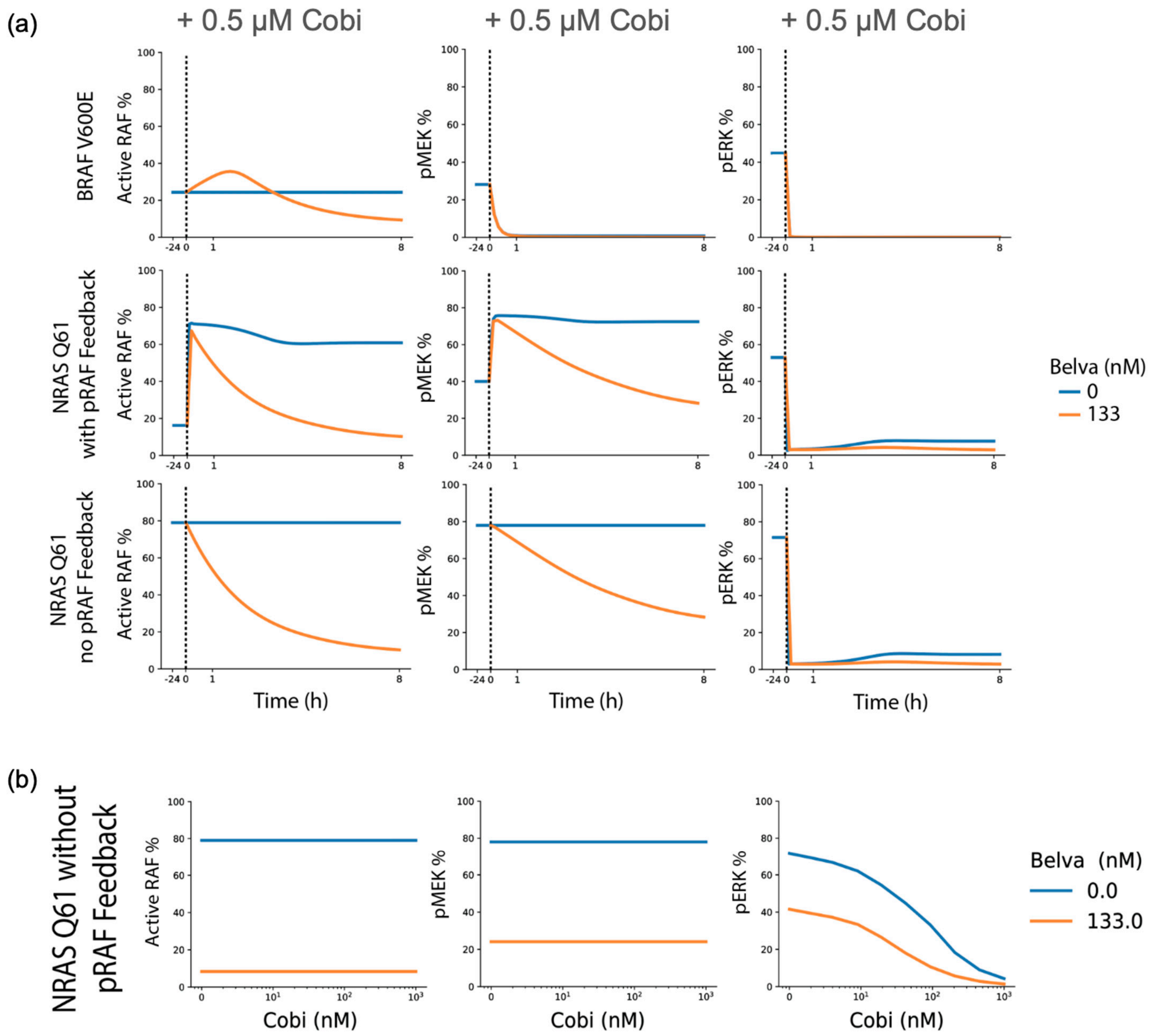

Figure S3. Additional model predictions. (a) Model predictions for percentages of active RAF, pMEK, and pERK over time. Models are initially in steady state without drug addition before being dosed with indicated Cobimetinib and Belva concentrations at t = 0. (b) Model predictions for steady state percentages of active RAF, pMEK, and pERK under indicated levels of Belvarafenib and Cobimetinib. Results are shown for NRAS Q61 model without pRAF feedback mechanism.

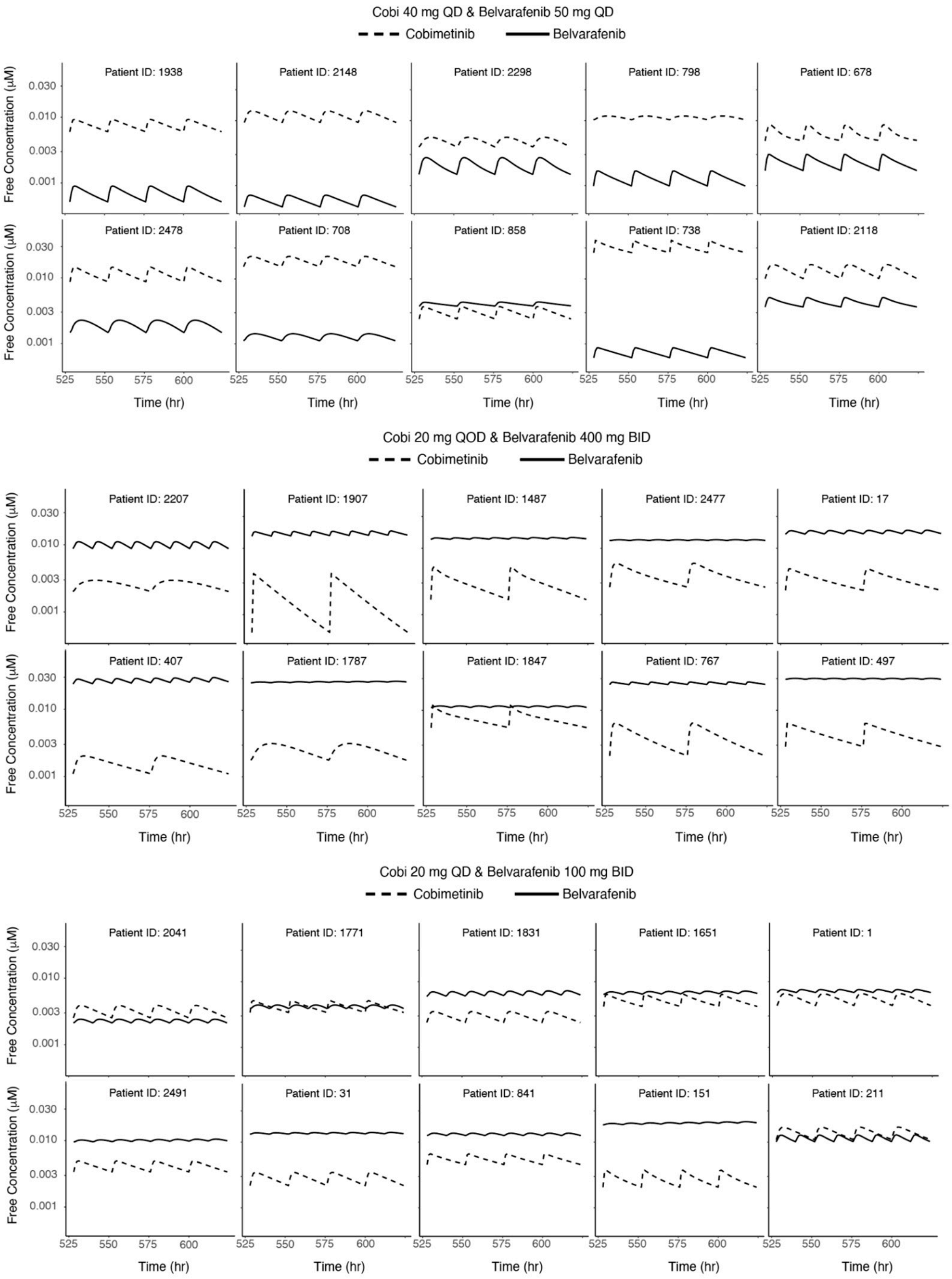

Figure S4. PK trajectories corresponding to the drug regimen and virtual patient combinations from fig 6. 96 hours are shown corresponding to at least two complete cycles of drug concentrations, the first 48 hours are used for the analysis shown in fig 6.

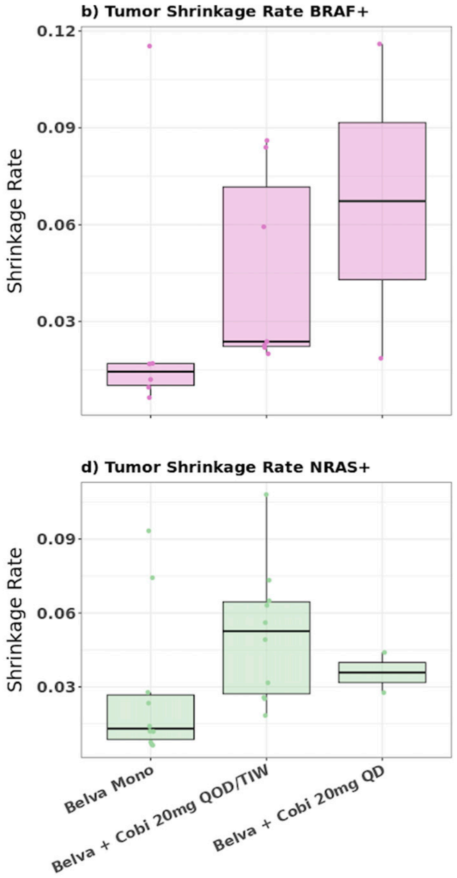

Figure S5. Simulations of shrinking rate of tumors in patients.
